# A top 5 list for French general practice

**DOI:** 10.1186/s12875-020-01235-5

**Published:** 2020-08-09

**Authors:** Agnès Hazard, Lucie Fournier, Louise Rossignol, Nathalie Pelletier Fleury, Corentin Hervé, Thibaud Pitel, Cécile Pino, Olivier Saint-Lary, Thomas Hanslik, Thierry Blanchon, Mathilde François

**Affiliations:** 1 Department of Family Medicine, Faculty of Health Sciences Simone Veil, University Versailles-Saint-Quentin-en-Yvelines, Villejuif, Paris, France; 2 Sorbonne Université, INSERM, Institut Pierre Louis d’Epidémiologie et de Santé Publique (IPLESP), Paris, France; 3 Centre for Research in Epidemiology and Population Health, French National Institute of Health and Medical Research (INSERM U 1018), University Versailles Saint-Quentin en Yvelines, University Paris-Sud, Villejuif, France; 4 AP-HP, Service de Médecine Interne, Hôpital Ambroise Paré, Boulogne Billancourt, France

**Keywords:** Medical overuse, General practice, France, Delphi method

## Abstract

**Background:**

Medical overuse is an issue that has recently gained attention. The “Choosing Wisely” campaign invited each specialty in each country to create its own top five lists of care procedures with a negative benefit-risk balance to promote dialogue between patients and physicians.

This study aims to create such a list for French general practice.

**Methods:**

A panel of general practitioners (GPs) suggested care procedures that they felt ought to be prescribed less. Using the Delphi method, a short list of those suggestions was selected. Systematic literature reviews were performed for each item on the short list. The results were presented to the panel to assist with the final selection of the top five list.

**Results:**

The panel included 40 GPs. The list includes: i/ antibiotics prescription for acute bronchitis, nasopharyngitis, otitis media with effusion, or uncomplicated influenza, ii/ systematic prostate specific antigen testing in men older than 50, iii/ prescription of cholinesterase inhibitors for mild cognitive impairment and for Alzheimer’s disease and memantine for Alzheimer’s disease, iv/ statins prescription in primary prevention of cardio-vascular risk in older patients, and v/ benzodiazepine or benzodiazepine-like agents prescription for generalised anxiety, insomnia, and for all indications in older patients.

**Conclusions:**

This study resulted in a French top five list in general practice using a panel of GPs. All the items selected have a negative risk-benefit balance and are frequently prescribed by French general practitioners. This list differs from other top five lists for general practice, reflecting the local medical culture.

## Background

Overuse is defined as ‘a healthcare service [that] is provided under circumstances in which its potential for harm exceeds the possible benefit’ [1]. This multifactorial phenomenon has become more important over recent years [2, 3].

The main issue of overuse is the harm it causes to patients. Overuse includes overdiagnosis and overtreatment, exposing patients to anxiety, pain, and discomfort stemming from unneeded tests and treatments [4, 5]. Secondarily, overuse leads to an unwarranted increase in healthcare spending. Overtreatment was estimated to represent at least 6% of the total US healthcare spending in 2011 [6].

*In order to limit medical overuse, campaigns such as ‘Choosing Wisely’ have been launched. The aim of this campaign is to encourage physicians and patients to* discuss unjustified and potentially harmful care procedures in order to reduce prescription rates. It is based on the creation of lists of five care procedures (treatment, tests, or procedures) that are commonly prescribed and which are not supported by evidence and are potentially harmful [7]. These lists, called top five lists, are specific to each medical specialty and to each country.

Top five lists are tools that can be used as part of wider campaigns to curb overuse. They can increase awareness of overuse among physicians and patients [8]. Over 60 medical specialties in 12 countries have created top five lists. In general practice, six countries have composed such a list: the United States [9], Canada [10], Switzerland [11], Australia [12], Italy [13], and the United Kingdom [14].

The existing top five list in general practice are all different, even when they come from bordering countries (e.g. Italy and Switzerland), mostly because they reflect the idiosyncrasies of each healthcare system and the local medical culture. Therefore, we felt that a French top five list in general practice was likely to differ from the previously published lists. The objective of this study was to create a French top five list in general practice.

## Methods

**Fig. 1:**
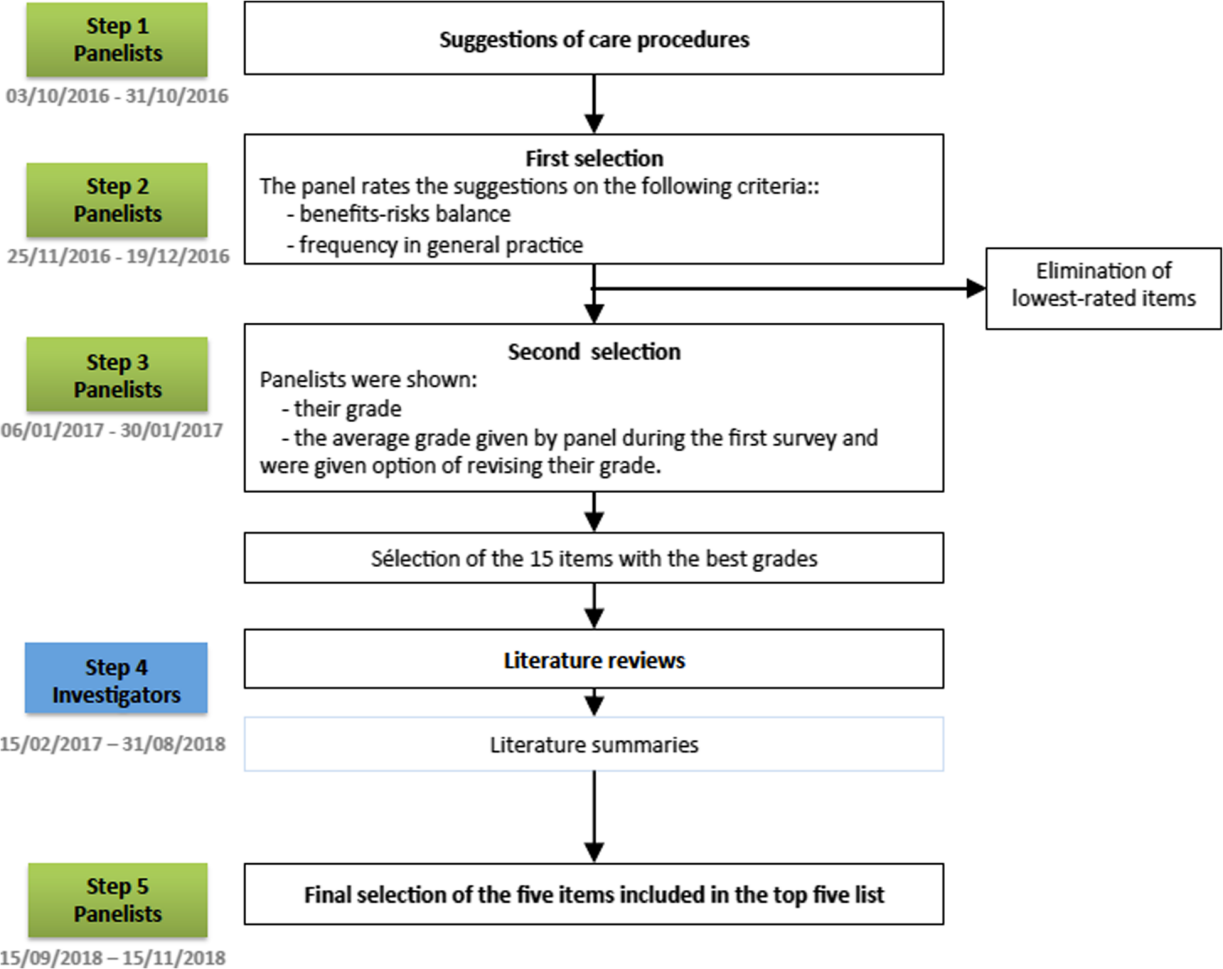
Visual representation of the study

The process used to create this top five list relied both on the participating general practitioners’ (GPs) expertise and on literature reviews. It was divided into five steps (Fig. 1). The protocol of this study was published elsewhere [15].

### Population

We elected to use a panel of GPs composed of non-academic GPs as well as GPs from university departments, in order to combine on-the-ground knowledge and academic expertise, and to ensure a diversity of opinions. The first recruitment source was the Sentinelles network, a research and surveillance network of GPs, located throughout mainland France [16]. Physicians participating in Sentinelles were presented with the protocol of the study and asked if they wished to participate. Among the GPs who volunteered, a sample was selected to ensure that the panel was diverse in terms of gender, age, location, rural/urban status, and practice size.

The second group of recruited GPs was composed of GPs belonging to general practice university departments and who had expertise in epidemiology and overuse. They were identified through the French National College of Teachers in General Practice.

At inclusion, GPs were asked to fill out a short descriptive questionnaire about their gender, age, location, and years of experience.

### Delphi method—list of fifteen items

For the first step, GPs in the panel were asked to submit suggestions of care procedures (tests, treatments, and procedures) that they felt ought to be prescribed less often, along with the indication for the procedure. These care procedures had to meet the following criteria: negative benefit-risk balance, commonly prescribed in general practice, and relevant to general practice. The suggestions were submitted anonymously using a Web application created specifically for the study.

The suggestions were then reformulated and regrouped by three investigators, when they were similar. If suggestions mentioned several care procedures (e.g. memantine and cholinesterase inhibitors for Alzheimer’s disease), we decided not to modify or split them. If this list contains more than 100 care procedures, only those cited twice were selected for the next step. This constituted the first list.

Using the Delphi method, the panel was asked twice to rate the items’ benefit-risk balance and frequency in general practice. During the two rounds, physicians had to vote for each indication for each procedure. During the second survey, panellists were shown their grade and the average grade given by the panel during the first survey, and were given the option of revising their grade.

Using the grades from the second survey, the fifteen most relevant procedures in terms of medical overuse were selected. The best rated indications/procedures pairs were selected until fifteen distinct procedures had been identified. Then the indications were grouped by procedure.

### Literature summary

For each of the fifteen items on the second list, literature reviews were carried out by following the methods used in the *Cochrane Handbook for Systematic Reviews of Interventions* [17]. The method used to carry out these literature reviews is detailed in Additional file 1. When recent, good quality meta-analysis or systematic review already existed, there was two possibility: first, if there were recent articles on the topic, an update was planned; second, if it was not possible to carry out an update, the best meta-analysis or systematic reviews were used. The best meta-analysis or systematic reviews were determined by the R-AMSTAR score [18]. The findings of these literature reviews were then synthesised to obtain summaries.

In the literature summaries, the efficacy and safety of the item was presented using pictograms representing respectively the number needed to treat and the number needed to harm. The short summaries also contained the following data: the current French recommendations on the item, data on the prevalence and incidence of the condition in France (if available), or worldwide, and on the cost to the French national health service, using publicly available data [19]. The short summaries were designed to be as homogenous as possible and aimed to be objective in their presentation of the literature. They were proofread by eight academic GPs for clarity and homogeneity.

### Final step

The fifteen literature summaries were presented to the GP panel. The GP panel was asked to select and then rank the five items that should be included in the top five list, using the Web interface. An item ranked first received 5 points, an item ranked second received 4 points, and so on. The points for each item were then added and the five items with the most points made up the top five list.

## Results

Forty GPs were recruited in the panel in September 2016: thirty from the French Sentinelles network (out of 51 volunteers) and ten from general practice university departments. Their characteristics are presented in Table 1.

**Table 1:** Characteristics of the general practitioners included in the panel

General practitioners		N	%	Mean (min-max)
**Sex**				
Male		28	70.0	
Female		12	30.0	
**Age**				
Age (in years)				47.8 (28–70)
Age group	[20–40]	16	40.0	
	[40–60]	16	40.0	
	[60–80]	8	20.0	
**Location**				
Île de France		15	37.5	
North-East		8	20.0	
North-West		7	17.5	
South-East		7	17.5	
South-West		3	7.5	
**Experience**				
Years since of start of practice				18 (2–43)
**Activity**				
Number of consultations per week				94 (31–200)
Number of half days worked per week				8.5 (5–12)
**Type of practice**				
Group practice		26	65.0	
Individual		14	35.0	
**Practice area**				
Urban		17	42.5	
Semi-rural		11	27.5	
Rural		12	30.0	

During the suggestion step, which took place in October 2016, a total of 346 items were suggested. Once reformulated, 166 items remained. After selection of the care procedures cited at least twice, 93 care procedures associated with their indications were therefore selected for the second step; they are presented in Additional file 1.

The participation rate of the panel for steps one to three was 100%.

The review of the fifteen suggestions began in February 2017 and ended in August 2018. For twelve items, meta-analyses or literature reviews of good quality were identified. The mean R-AMSTAR grade for the meta-analyses selected was 36. For three indications of three items (Non-steroidal anti-inflammatory agent for pharyngitis, antibiotic for uncomplicated influenza and Tramadol in the elderly), no suitable meta-analyses or literature reviews were identified. Due to various reasons (low quality of studies, lack of sufficient statistical data, lack of common end point between the studies, etc.), the investigators did not carry out new meta-analyses. For these three indications, the results of individual studies were presented. Three of this reviews and their abstract have been published [20–22]. A sample summary is available in the Additional file 1. The studies selected are presented in Additional file 1.

The final selection by the panel occurred in September and October 2018. The participation rate was 87.5%. The fifteen items were given grades ranging from 9 to 88, with a mean grade of 35. The selected items, constituting the French top five list in general practice, are presented in Table 2.

**Table 2:** List of the fifteen items obtained after the second Delphi vote

Care procedure	Indications	Grade given by the panel during the final step
Prescription of antibiotics^a^	Acute bronchitis, nasopharyngitis, otitis media with effusion, uncomplicated influenza	88
Prostate Specific Antigen (PSA) testing^a^	In systematic screening of prostate cancer in men older than 50, with no information given to the patient regarding the benefits and risks	69
Prescription of cholinesterase inhibitors and memantine^a^	Mild cognitive impairment and Alzheimer’s disease	62
Prescription of statins^a^	Primary prevention of cardio-vascular risk in older patients	43
Prescription of benzodiazepine and benzodiazepine-like agents ^a^	For insomnia	38
	For generalised anxiety	
	In older patients, for all indications	
Prescription of Dipeptidyl Peptidase-4 inhibitors (DPP-4 inhibitors)	Type 2 diabetes	36
Prescription of a homeopathic treatment (Influenzinum)	Flu prevention	35
Prescription of antitussive or expectorants agents	For acute cough or acute bronchitis care	28
Long-term prescription of proton pump inhibitors	Without reviewing the indication	28
Prescription of vasodilator agents	Peripheral Arterial Disease	23
Prescription of tramadol or tramadol with paracetamol	Pain care in older patients	20
Prescription of lumbar scanner	Low back pain evolving for less than 6 weeks	16
Prescription of allopurinol	Asymptomatic hyperuricemia in prevention of gout attack, renal hypertension, cardio-vascular disease	16
Prescription of a non-steroidal anti-inflammatory agent (NSAID)	Symptomatic treatment of acute sinusitis and pharyngitis	14
Mammography	Systematic screening for breast cancer in women with no information given to the patient regarding the benefits and risks	9

^a^Included in the Top 5 list

## Discussion

This study resulted in a French top five list in general practice using a panel of GPs. The five care procedures selected were: i/ antibiotics prescription for acute bronchitis, nasopharyngitis, otitis media with effusion, or uncomplicated influenza, ii/ systematic prostate specific antigen (PSA) testing in men older than 50, iii/ prescription of cholinesterase inhibitors for mild cognitive impairment and for Alzheimer’s disease and memantine for Alzheimer’s disease, iv/ statins prescription in primary prevention of cardio-vascular risk in older patients, and v/ benzodiazepine or benzodiazepine-like agents prescription for generalised anxiety, insomnia, and for all indications in older patients. This list differs from other top five lists for general practice [9–14].

Among the five items included in this top five list, three were similar to items present in at least one other general practice list [9–13]. The item about the prescription of antibiotics is similar to the item ‘antibiotics for upper respiratory infections with no signs of gravity’, found in four out of six of the existing lists [9–11, 13]. The item about the dosage of PSA is similar to the item ‘systematic prostate cancer screening in asymptomatic men over 50 with no information on the benefits and risks’, found in the Swiss top 5 list [11]. The item about benzodiazepine prescription is close to Australian and Italian items [12, 13]: the Australian list advised against prescribing benzodiazepines to patients with a history of substance misuse (including alcohol) or multiple psychoactive drug use; the Italian list was broader in its recommendations and advised against routinely prescribing benzodiazepines or Z-drugs in elderly patients to treat insomnia.

The inclusion of these three items is consistent in the French context, especially in general practice. Firstly, the reduction of unnecessary antibiotic prescription is an important public health issue in France, where antibiotic prescriptions remain high and most antibiotics (70%) are prescribed by GPs [23]. Despite public health campaigns antibiotic prescribing has increased again in recent years [23, 24]. Secondly, between 2013 and 2015, 48% of French men over 40 had at least one PSA assay; this percentage rose to 90% when considering men aged between 65 and 79 [25]. Most of these tests (88%) were prescribed by a GP [26]. Nearly half of French GPs are still convinced of the effectiveness of PSA and 65% regularly prescribe it [27]. Thirdly, France is the second largest consumer of benzodiazepines in Europe. In 2015, 13.4% of the population had at least one prescription of benzodiazepines and 90% of benzodiazepines were prescribed by GPs [28].

Two items on our list were not found on other general practice top five lists: ‘cholinesterase inhibitors and memantine for mild cognitive disorder and Alzheimer’s disease’ and ‘statins in primary prevention for cardiovascular diseases in older patients’. It is not surprising to find these items in the top 5 list. Cholinesterase inhibitors and memantine came under the spotlight in medical news in 2018 when the reimbursement of these treatments by the French health insurance system ended in the summer [29]. Although the prescription of cholinesterase inhibitors and memantine decreased in France as of 2011, their prescription remains significant: the prescription cost for the French health insurance system was nearly €58 million in 2017 [30]. Lastly, the official French guidelines recommended evaluating a patient’s risk of a cardiovascular event using the SCORE risk charts [31]. However, these charts have not been evaluated for patients older than 65. In France, in 2014, a lipid-lowering drug was prescribed to 39% of people aged between 65 and 84 [32]. It should also be noted that GPs participating in the Sentinelles network were invited to participate in the SITE study, a study on statins cessation in elderly patients [33].

The motivations of top five list creators have also been questioned, as it has been noted that medical societies often included other specialty services in their top five list and did not include revenue-generating services whose value is questionable [34]. All the items in our top five list are mostly prescribed by general practitioners. In France, most GPs are paid according to the number of consultations they conduct and for the most part their earnings are not dependent on the treatment they prescribe. Therefore, we feel that financial self-interest did not play an important role in the selection of the items included in our top five list.

### Limitations and strengths

This study presented some limitations. Firstly, the GPs included in our study already had opinions shaped by their experiences and public discourse about what constitutes overuse. To limit the weight of their biases, they were presented with literature summaries. Secondly, this list was compiled using a panel of GPs—no patients were consulted. This is a weakness because patients are important actors in overuse. Thirdly, investigators reformulated suggestions made by the panel to make them clearer and more homogeneous. It is possible that the investigators misunderstood some suggestions and inadvertently changed their meaning when reformulating. Finally, for 3 indications (Non-steroidal anti-inflammatory agent for pharyngitis, antibiotic for uncomplicated influenza and Tramadol in the elderly), no systematic review was available and none could be conducted due to the lack of data and the low quality of the studies found for these three subjects. The data provided to GPs on these 3 subjects are therefore not of sufficient quality, but these data were preferred over no data at all.

This study also had some strengths. To establish this list, GPs used scientific data specifically provided to them in summarized form by the research team. This is unique in the context of the development of top five lists for general practice, both in terms of the methods used to obtain it and in its content. All the items selected have, according to the data in the literature, a negative risk-benefit balance, and the frequency of their prescriptions in general practice is confirmed by data (when available). Our panel of GPs was recruited from two populations of GPs— academics and non-academics—, in order to ensure that different perspectives on overuse were included in the panel [35]. Given that no recommendation exists on the optimal size of the Delphi panel, we elected to consult a relatively small panel of GPs to create this list, instead of trying to collect the opinions of as many GPs as possible. We felt that relying on a relatively small group of motivated participants would guarantee better participation. This strategy was successful given that the participation was 100% for the early steps and 87.5% for the last step. The Delphi method was used to compile a list of fifteen items to be reviewed. This method is commonly used in the creation of top five lists [11, 36]. It presents many advantages; notably, it allows a group to reach a consensus without being unduly influenced by a few assertive members and it can be carried out remotely, facilitating the participation of geographically diverse participants.

## Conclusion

This study resulted in a top 5 list for French general practice, compiled with the help of a panel of French GPs. The results reflect the specificity of the French health system and the idiosyncrasies of local medical culture. However, we feel that in order for these campaigns to be effective in curbing overuse, patients need to be included and heard. That is why the next phase of this project will aim to adapt the fifteen summaries of the literature for patients and ask a sample of French patients to choose their own top 5 using the summaries.

## Supplementary information

**Additional file 1: Appendix 1.** The method used to carry out literature reviews. **Appendix 2.** 93 care procedures associated with their indications. **Appendix 3.** A sample summary. **Appendix 4.** Studies selected for summaries.

## Data Availability

All the data of this work are accessible on simple request to the authors.

## Abbreviations

AMSTAR: A Measurement Tool to Assess Systematic Reviews
GPs: General Practitioners
PSA: Prostate Specific Antigen
SCORE: Systematic COronary Risk Evaluation
SITE: Statins in the elderly
US: United States
