# Supplementary material for: A top 5 list for French general practice
Source: BMC Fam Pract. 2020 Aug 9;21:161. doi: 10.1186/s12875-020-01235-5 (PMC7416409; doi:10.1186/s12875-020-01235-5)
Supplement: Supplementary file 1 — Additional file 1: Appendix 1. The method used to carry out literature reviews. Appendix 2. 93 care procedures associated with their indications. Appendix 3. A sample summary. Appendix 4. Studies selected for summaries. [file 12875_2020_1235_MOESM1_ESM.docx]

**Appendix 1**: The method used to carry out literature reviews

1-1: General process

For each item the review question was defined by specifying the population, intervention, comparison and outcome (PICO). Search equations for MedLine, Embase and the Cochrane Library were devised with the help of an academic librarian specialised in medical sciences. Articles were selected based on the title and the abstract by two investigators working independently. Conflicts regarding the inclusion of a given article were resolved by a discussion between the two investigators. Each investigator analysed the quality and the risk of bias of the included articles independently using standardised evaluation tools: STROBE, CONSORT and R-AMSTAR for cohort studies, clinical trials and systematic reviews respectively. When several recent systematic reviews or meta-analyses were found, the studies with the highest R-Amstar score were used. In the absence of recent systematic reviews or meta-analyses, it was examined whether one could be done. Otherwise, there were plans to select the clinical trials and cohort studies as a last resort.

1-2: Flow chart of the 15 literature reviews


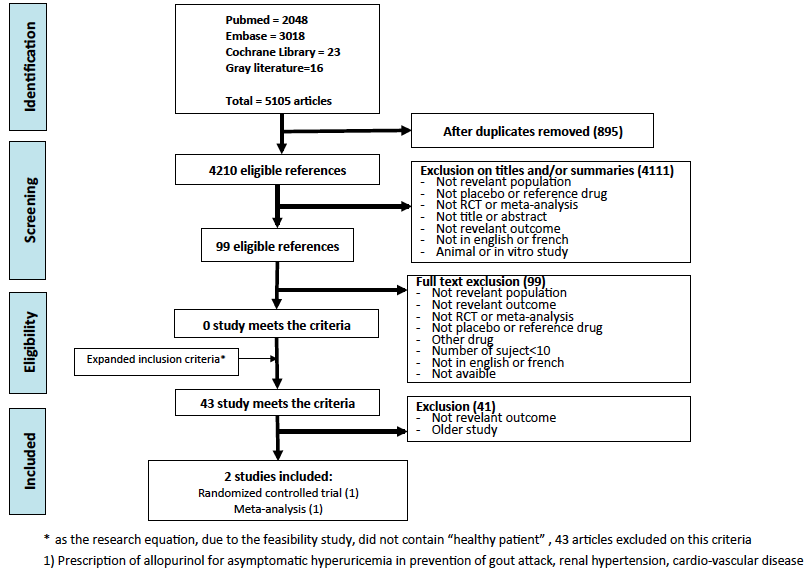


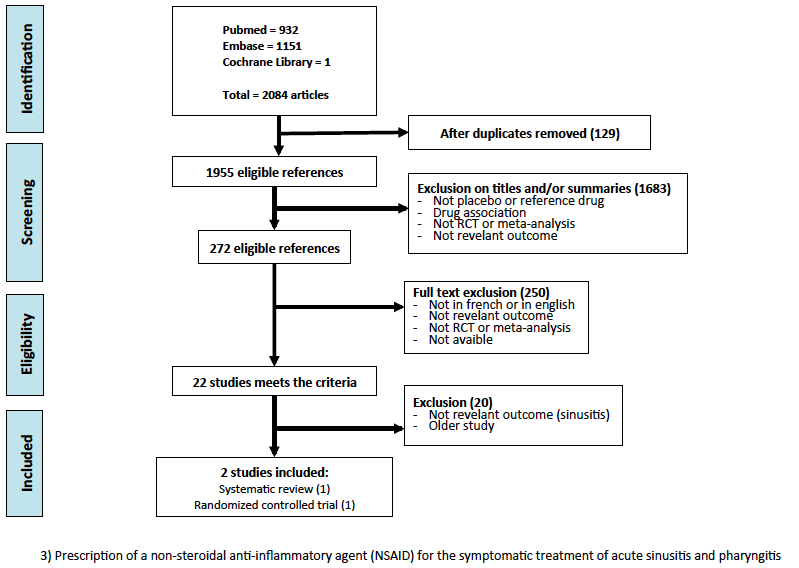

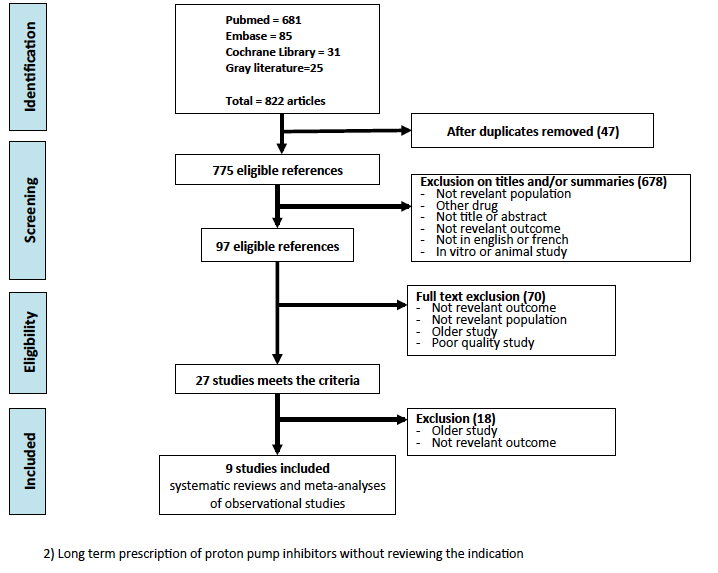


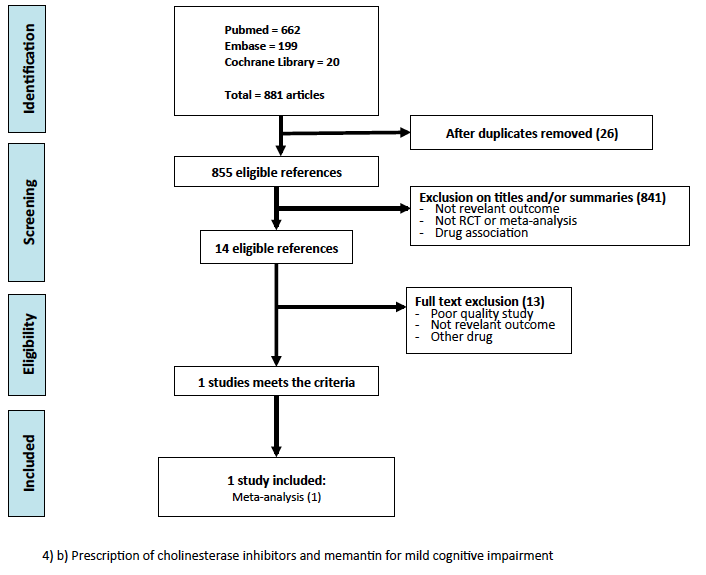

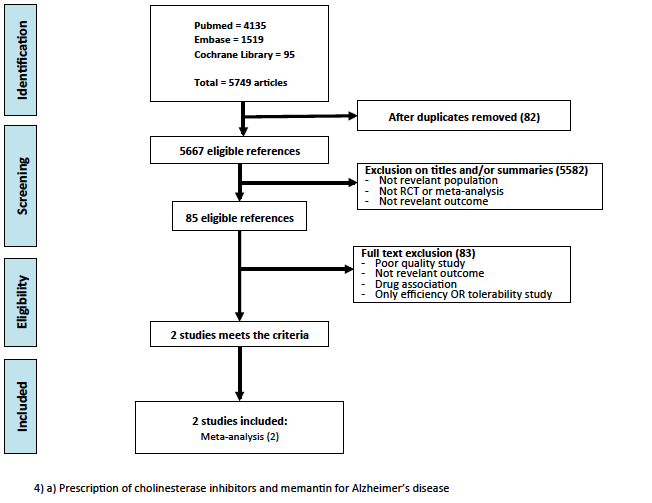


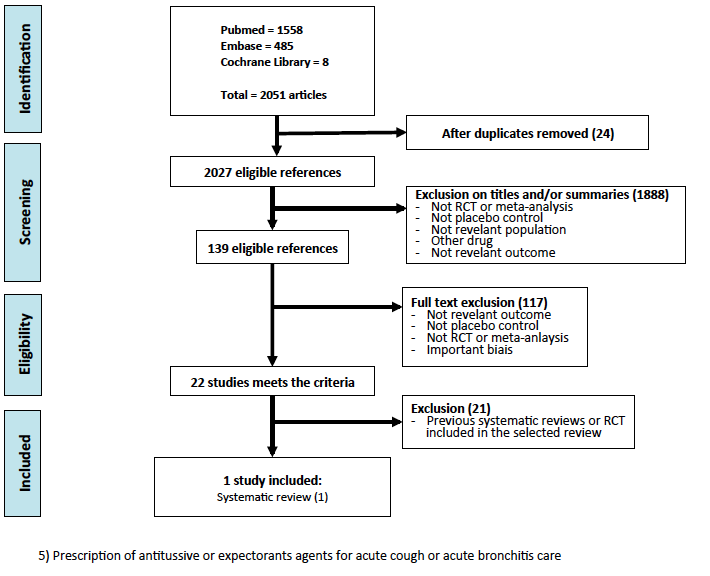

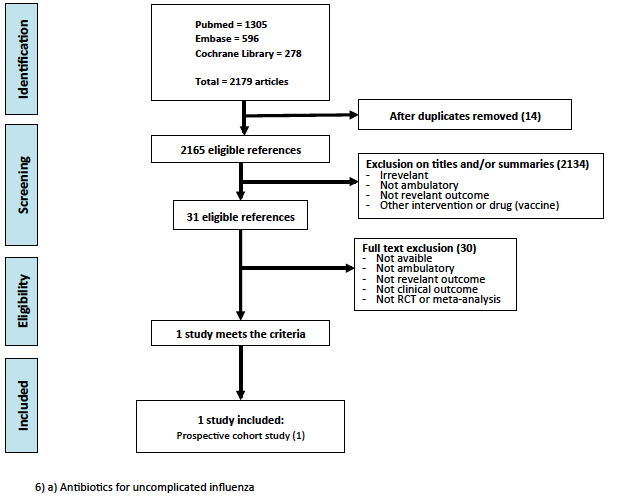


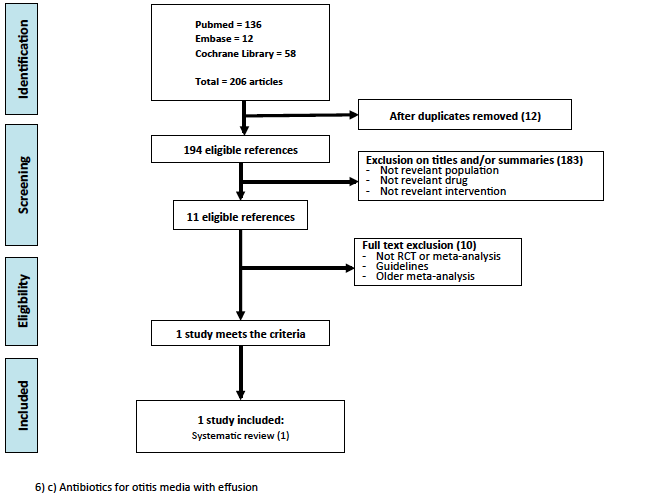

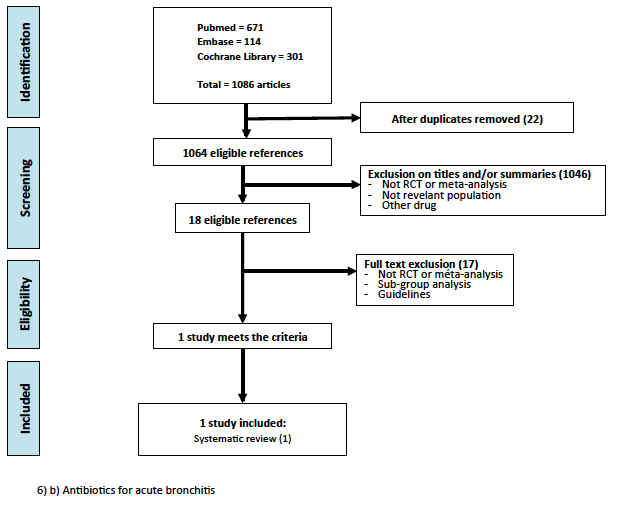


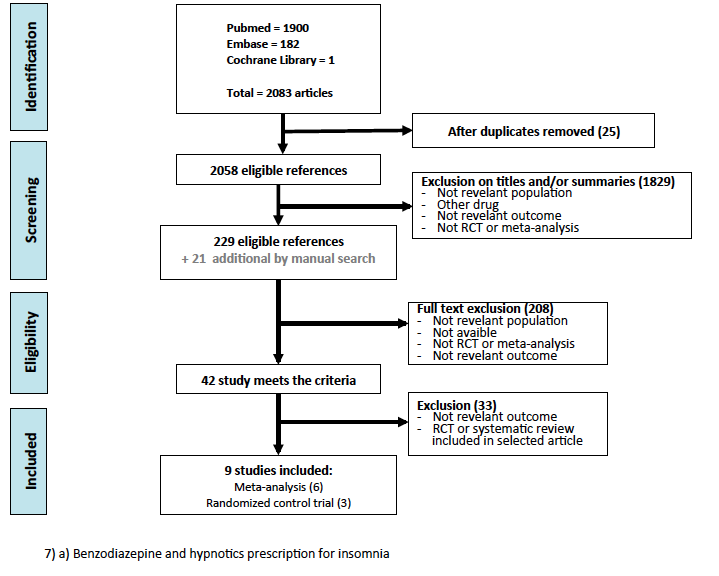

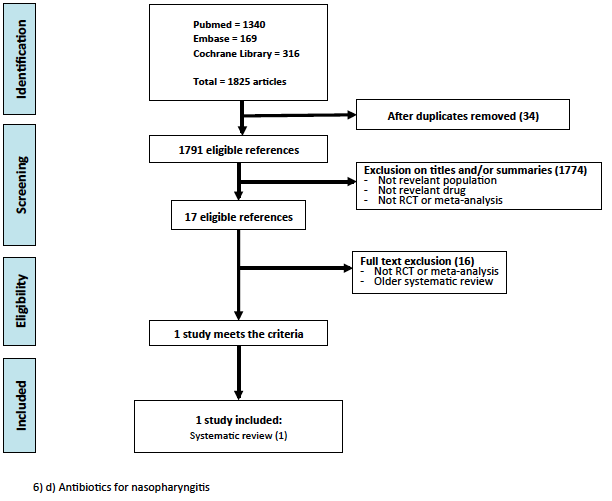


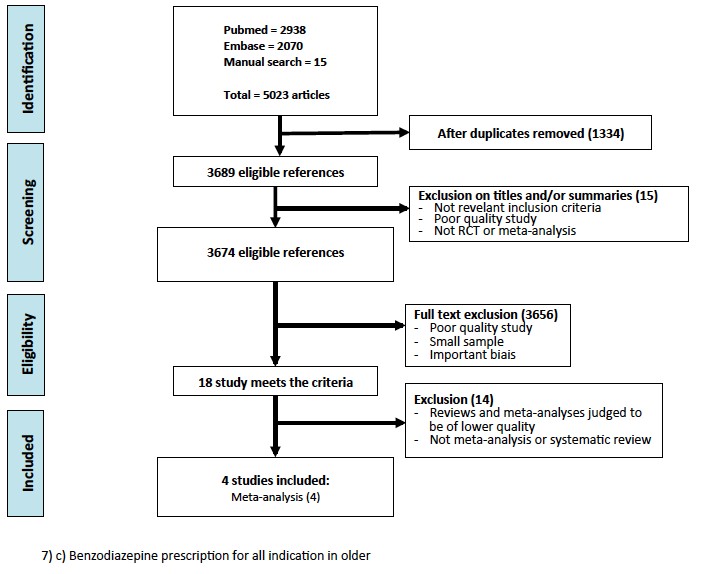

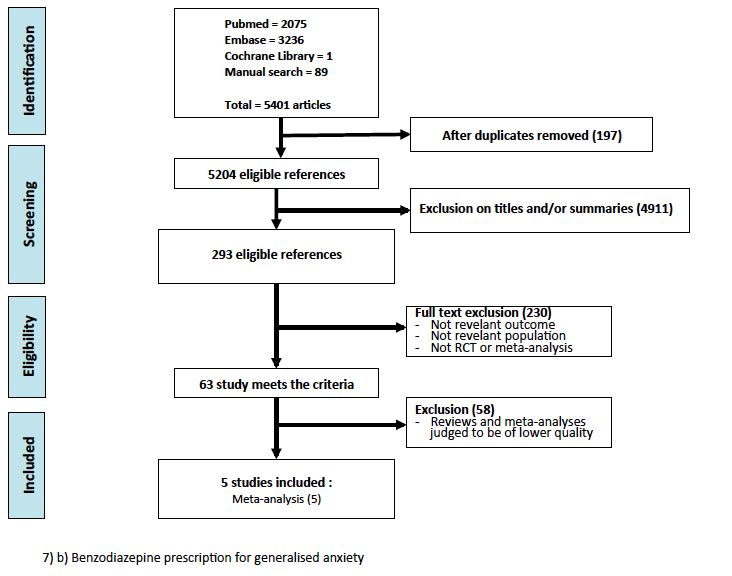


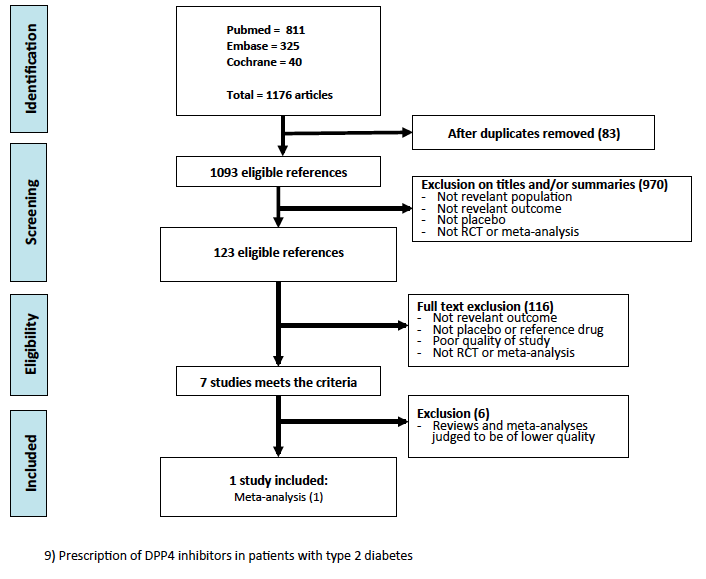

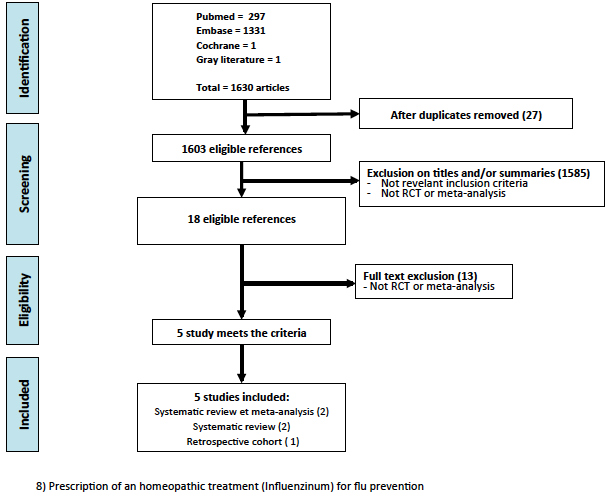


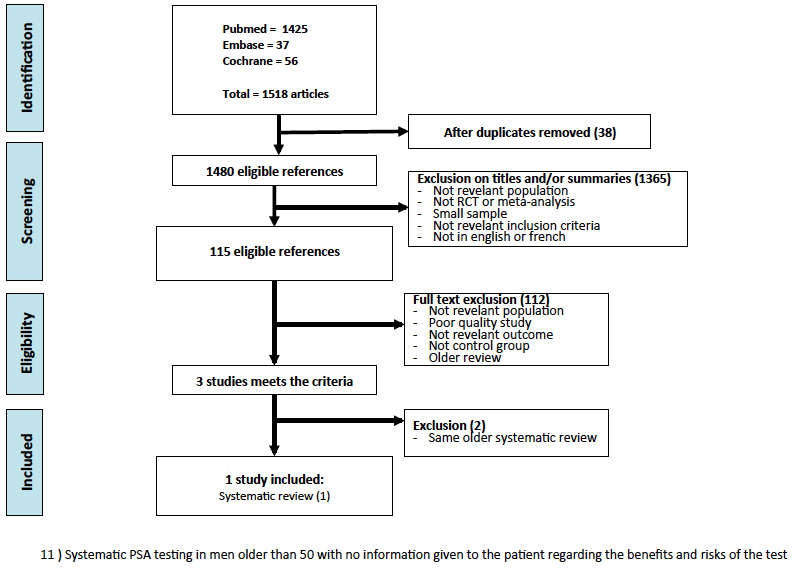

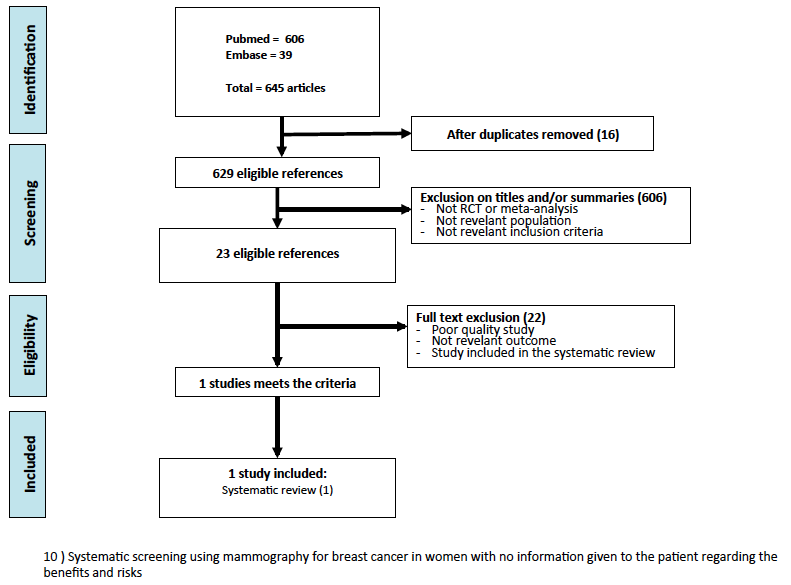


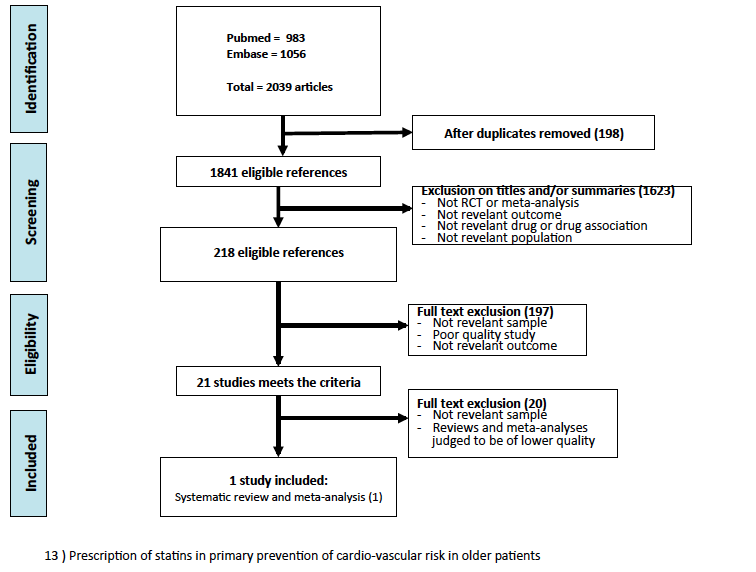

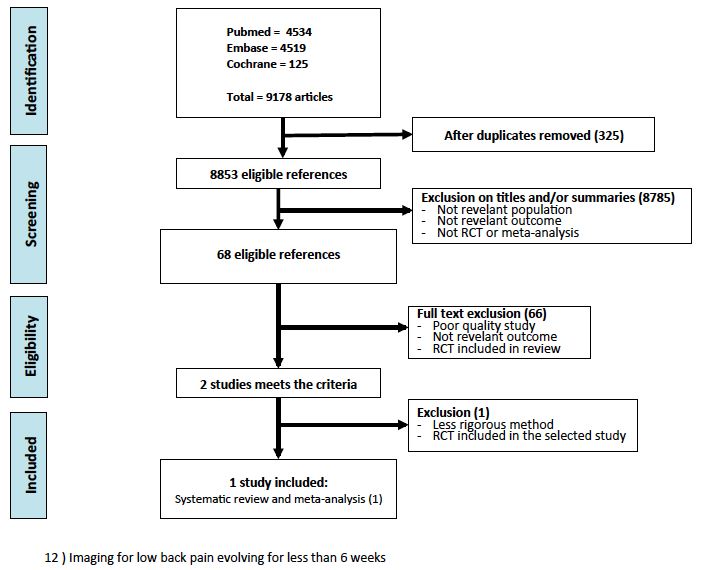


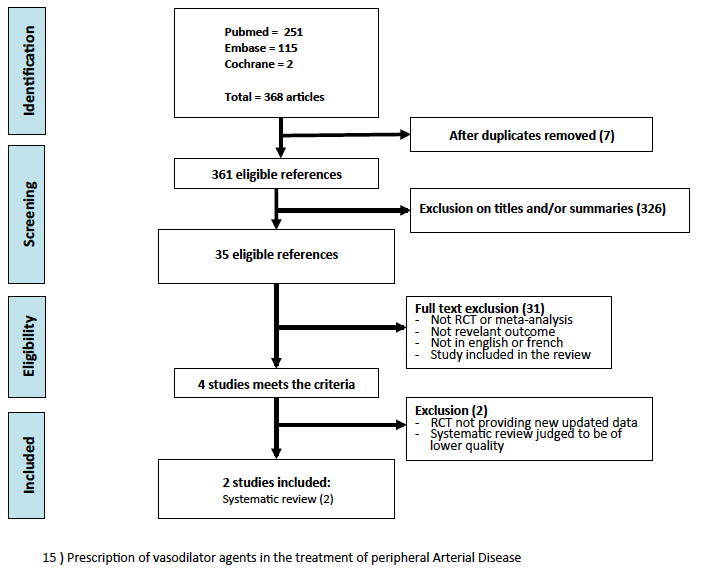

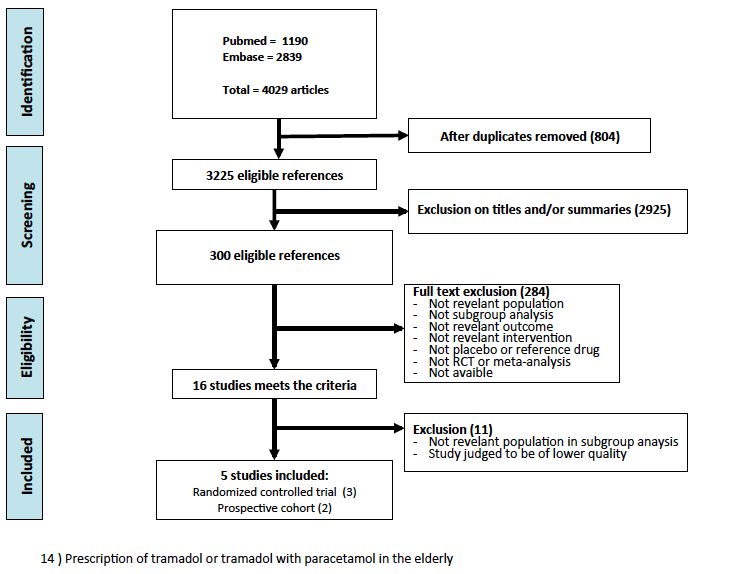


**Appendix 2**: 93 care procedures associated with their indications

| Item | | Indication 1 | | Indication 2 | | Indication 3 | | Indication 4 | | Indication 5 | | Indication 6 | | Indication 7 | | Indication 8 | | Indication 9 | | Indication 10 | | Indication 11 |
| --- | --- | --- | --- | --- | --- | --- | --- | --- | --- | --- | --- | --- | --- | --- | --- | --- | --- | --- | --- | --- | --- | --- |
| PSA testing | | systematic prostate cancer screening for primary prevention | | systematic prostate cancer screening for men older than 50 | | systematic prostate cancer screening without informing the patient of the benefits and risk | |  | |  | |  | |  | |  | |  | |  | |  |
| X-ray of the lower back | | Back pain | | sciatica | |  | |  | |  | |  | |  | |  | |  | |  | |  |
| Insulin prescription | | Type 2 diabetes | | Type 2 diabetes in patients with a BMI > 30 kg/m² | |  | |  | |  | |  | |  | |  | |  | |  | |  |
| Prescription of antibiotics | | nasopharyngitis | | Tonsillitis | | Tonsilitis with erythematous pulpy and without Rapid strep test | | acute otitis media | | acute otitis media in children older than 2 years old without severe symptoms | | otitis media with effusion | | Bronchitis | | Influenza with no secondary infection | | Patient expectation | | sinusitis | | Viral otolaryngologic infection with no sign of secondary bacterian infection |
| Prescription of local antibiotics | | Acute otitis media | | Viral conjunctivitis | |  | |  | |  | |  | |  | |  | |  | |  | |  |
| Antivertigo drugs (TANGANYL, AGYRAX) | | vertigo treatment | |  | |  | |  | |  | |  | |  | |  | |  | |  | |  |
| Aspirin | | primary prevention of cardiovascular diseases | | Primary prevention of cardiovascular diseases in low-risk patients | | Viral infection in patients under 18 years old | |  | |  | |  | |  | |  | |  | |  | |  |
| Ezetimibe | | primary prevention of cardiovascular diseases | |  | |  | |  | |  | |  | |  | |  | |  | |  | |  |
| Statins | | primary prevention of cardiovascular diseases | | Primary prevention of cardiovascular diseases in low-risk patients | | For older patients in secondary prevention | | For older patient in primary prevention | |  | |  | |  | |  | |  | |  | |  |
| Fribrate | primary prevention of cardiovascular diseases | | [other than gemfibrozil](https://www.hetop.eu/hetop/en/?rr=MSH_D_015248&q=MSH_D_015248) | |  | |  | |  | |  | |  | |  | |  | |  | |  |  |
| HbA1c testing more than once a year | | Type 2 diabetes | |  | |  | |  | |  | |  | |  | |  | |  | |  | |  |
| TSH testing | | systematically for asthenia | | Hypothyroid screening in asymptomatic patient | | more than once a year in a treated patient with normal tests | |  | |  | |  | |  | |  | |  | |  | |  |
| Lab tests | | Patient demand | | with no clinical justification | |  | |  | |  | |  | |  | |  | |  | |  | |  |
| Serum iron testing | | Associated with ferritin | | anemia | |  | |  | |  | |  | |  | |  | |  | |  | |  |
| Urea testing | | associated with creatinine testing | | associated with creatinine testing when monitoring renal fonction | | systematically | | without urine test | |  | |  | |  | |  | |  | |  | |  |
| prescription of antitussive agents | | Viral otolaryngology infection | | wet cough | | dry cough | | minor cough | |  | |  | |  | |  | |  | |  | |  |
| Prescription of corticosteroid nasal spray | | Rhinitis | | nasopharyngitis | | sinusitis | |  | |  | |  | |  | |  | |  | |  | |  |
| Prescription of corticosteroid pills | | nasopharyngitis | | sinusitis | | laryngitis | | acute laryngitis | | tonsillitis | | otitis | |  | |  | |  | |  | |  |
| topical nsaids | | massages | | Degenerative disc disease of the lower back | |  | |  | |  | |  | |  | |  | |  | |  | |  |
| Prescription of physical therapy | | Osteoarthritis | | Fibromyaligie | | long term, for older patients with osteoarthritis | |  | |  | |  | |  | |  | |  | |  | |  |
| Presciption of respiratory physical therapy | | newborn bronchiolitis | | Bronchiolitis | | Bronchiolitis without respiratory cmorbidities | |  | |  | |  | |  | |  | |  | |  | |  |
| vitamin D testing | | Systematic vitamin D deficiency screening in the general population | | Systematic vitamin D deficiency screening in older patients | | asthenia | | discomfort | | diffuse pain | |  | |  | |  | |  | |  | |  |
| Prescription of vitamin D | | diffuse pain | | general population | | asthenia | |  | |  | |  | |  | |  | |  | |  | |  |
| Homeopathy | | All indications | | flu prevention (influenzinum) | | Flu treatment | | trauma (arnica) | |  | |  | |  | |  | |  | |  | |  |
| Lipid abnormality assesment | | Systematic screening | | Yearly systematic screening | | Screening in a patient with no other cardiovascular risk factors | | Screening in older patients | | Screening in patients already treated by statins in primary prevention | |  | |  | |  | |  | |  | |  |
| Cholesterol testing | | primary prevention | | primary prevention in patients older than 80 | | Yearly monitoring if the last recent (<3 years) test was normal | | More than once a year | | yearly for patients taking the highest statin dosage | |  | |  | |  | |  | |  | |  |
| Prescription of hypnotics | | patients older than 70 | | sleep disorder | |  | |  | |  | |  | |  | |  | |  | |  | |  |
| Chest CT scan | | Patients with normal CT scan in the last 3 months | | Asbestosis | |  | |  | |  | |  | |  | |  | |  | |  | |  |
| Prescription of medical leave longer than 2 weeks | | Anxiety and depression | | conflict at work | |  | |  | |  | |  | |  | |  | |  | |  | |  |
| Knee MRI | | Articular disk lesions | | without an indication that could lead to surgery | | as a first step for articular knee pain | |  | |  | |  | |  | |  | |  | |  | |  |
| Prescription of mucolytic | | Bronchitis | |  | |  | |  | |  | |  | |  | |  | |  | |  | |  |
| Prescription of proton pump inhibitors | | Children with gastroesophageal reflux | | Gastroesophageal reflux | | Systematic prevention when prescribing antiplatelet drugs | | Systematic prevention when prescribing NSAIDs | |  | |  | |  | |  | |  | |  | |  |
| Long term proton pump inhibitors prescription | | Epigastrium pain | | dyspepsia | | with no revision of the indication | | without therapeutic window | |  | |  | |  | |  | |  | |  | |  |
| Systematic electrocardiography | | Medical certificate stating that there is no contraindication to sport | | Postoperative follow-up | |  | |  | |  | |  | |  | |  | |  | |  | |  |
| Medical certificate stating that there is no contraindication to sport | | sport practiced in a sports club | | when it's not required | |  | |  | |  | |  | |  | |  | |  | |  | |  |
| Thyroid ultrasound | | Systematically following the discovery of thyroid dysfunction | | Systematically in the follow up of thyroid dysfunctions | | Systematic screening of a thyroid cancer in an asymptomatic patient | | Cheking bening nodules | |  | |  | |  | |  | |  | |  | |  |
| Abdominal x-ray | | constipation | | all indications | |  | |  | |  | |  | |  | |  | |  | |  | |  |
| Chest x-ray | | Cancer screening for smokers | | asymptomatic patient | | lingering cough | | bronchitis | | pneumopathy | | exposure to asbestos | | for nurses at hiring | |  | |  | |  | |  |
| ankle x-ray | | outside of the Ottawa ankle rules | |  | |  | |  | |  | |  | |  | |  | |  | |  | |  |
| Prescription of a lumbar belt | | chronic back pain | | acute back pain | |  | |  | |  | |  | |  | |  | |  | |  | |  |
| Prescription of a muscle relaxant | | acute back pain | |  | |  | |  | |  | |  | |  | |  | |  | |  | |  |
| bone density test | | Osteoporosis screning in patient with no risk factors for osteoporosis | | Osteoporosis screening in patients with low risk | | Osteoporosis screening in post-menoposal women with no bone fracture antecedents | | osteoporosis monitoring | | in post menoposal women | | all indication in adults | |  | |  | |  | |  | |  |
| Prescription of angiotensin II receptor blockers | | hypertension | | hypertension or heart failure with prior prescription of an ACE inhibitor | |  | |  | |  | |  | |  | |  | |  | |  | |  |
| prescription erythrocyte sedimentation rate test | | all indications | | along with C-reactive protein | | inflammation assessment | |  | |  | |  | |  | |  | |  | |  | |  |
| Ibuprofen | | throat pain | | hyperthermia in children | | as a first intention before paracetamol | |  | |  | |  | |  | |  | |  | |  | |  |
| NSAID | | sinusitis | | tonsillitis | |  | |  | |  | |  | |  | |  | |  | |  | |  |
| Self-Monitoring of Blood Glucose | | type 2 diabetes | | type 2 diabetes when prescribing insulin is not considered | |  | |  | |  | |  | |  | |  | |  | |  | |  |
| Pap test prescription | | for female patients younger than 25 | | more often than every 3 years | | systematic screening | |  | |  | |  | |  | |  | |  | |  | |  |
| Prescription of a urine culture test | | post-infection monitoring | | uncomplicated cystisis | | uncomplicated and not recurrent cystitis | |  | |  | |  | |  | |  | |  | |  | |  |
| Doppler ultrasonography for suspected deep vein thrombosis | | in the monitoring of a slight to moderate chronic venous insufficiency | |  | |  | |  | |  | |  | |  | |  | |  | |  | |  |
| prescription of an antidiarrhoeal | | in non profuse diarrhea | | for children with gastroenteritis | | gastroenteritis | |  | |  | |  | |  | |  | |  | |  | |  |
| Hypnotics | | Occasional insomnia | | sleep disorders | |  | |  | |  | |  | |  | |  | |  | |  | |  |
| tumor marker testing | | cancer screening | |  | |  | |  | |  | |  | |  | |  | |  | |  | |  |
| uric acid testing | | asymptomatic patient | |  | |  | |  | |  | |  | |  | |  | |  | |  | |  |
| Phosphodiesterase inhibitor prescription | | spasmodic abdominal pain | |  | |  | |  | |  | |  | |  | |  | |  | |  | |  |
| paranasal sinus x-ray | | all indications | | chronic sinusitis | |  | |  | |  | |  | |  | |  | |  | |  | |  |
| Sulfonylurea prescription | | type 2 diabetes | |  | |  | |  | |  | |  | |  | |  | |  | |  | |  |
| DPP4 inhibitors prescription | | type 2 diabetes | |  | |  | |  | |  | |  | |  | |  | |  | |  | |  |
| Alpha-glucosidase inhibitor prescription | | type 2 diabetes | |  | |  | |  | |  | |  | |  | |  | |  | |  | |  |
| antidepressant presription | | depression of a slight to moderate intensity | | chronic anxiety | | work related suffering with no major impact on daily life | |  | |  | |  | |  | |  | |  | |  | |  |
| long term antidepressant prescription | | depression following a traumatic | | for more than 5 years | |  | |  | |  | |  | |  | |  | |  | |  | |  |
| Allopurinol | | asymptomatic hyperuricemia | |  | |  | |  | |  | |  | |  | |  | |  | |  | |  |
| Levothyroxine prescription | | To slow the growth of benign thyroid nodules | | subclinical hypothyroidism | |  | |  | |  | |  | |  | |  | |  | |  | |  |
| Mammography | | Breast cancer screening | | Breast cancer screening for women younger than 50 years | | Breast cancer screening for women from 50 to 74 years old | | Breast cancer screening for women older than 75 years | | breast cancer screening in a female patient who has not been informed of the benefits and risks | |  | |  | |  | |  | |  | |  |
| cholinesterase inhibitor | | Alzheiùer's disease | | moderate or severe cognitive disorders | |  | |  | |  | |  | |  | |  | |  | |  | |  |
| memantine | | maladie d'Alzheimer | |  | |  | |  | |  | |  | |  | |  | |  | |  | |  |
| Benzodiazepine | | sleep disorders | | with long half life for all indications | |  | |  | |  | |  | |  | |  | |  | |  | |  |
| Long term benzodiazepine | | for longer than 3 month for insomnia | | chronic anxiety | | all indications | |  | |  | |  | |  | |  | |  | |  | |  |
| anti-hpv vaccination | | systematically for cervical cancer prevention | |  | |  | |  | |  | |  | |  | |  | |  | |  | |  |
| Antihypertensive drug | | blood pressure lower than 160/100 in a patient with a low cardiovascular risk | |  | |  | |  | |  | |  | |  | |  | |  | |  | |  |
| lower back CT-scan | | without an indication that could lead to surgery and in order to find a Spinal disc herniation | | back pain | | back pain with fewer than 6 weeks of ineffective treatment | |  | |  | |  | |  | |  | |  | |  | |  |
| lower back MRI | | Nonspecific back pain | | back pain with fewer than 6 weeks of ineffective treatment | | lower back pain | |  | |  | |  | |  | |  | |  | |  | |  |
| rotavirus vaccination | | in children | |  | |  | |  | |  | |  | |  | |  | |  | |  | |  |
| Amiodarone | | for atrial fibrillation in older patients | |  | |  | |  | |  | |  | |  | |  | |  | |  | |  |
| D-dimer testing | | screening for deep vein thrombosis for patients over 70 years old | | to detect a pulmonary embolism | |  | |  | |  | |  | |  | |  | |  | |  | |  |
| Alkaline phosphatase | | when testing liver function | | Systematically | |  | |  | |  | |  | |  | |  | |  | |  | |  |
| Lyme disease serology | | due to patient demand | | for an erythema migrans with known tick bite | | after a tick bite with no erythema migrans | |  | |  | |  | |  | |  | |  | |  | |  |
| T3 and T4 testing | | systematically when testing TSH | |  | |  | |  | |  | |  | |  | |  | |  | |  | |  |
| Phlebotonics | | Chronic venous insufficiency | | hemorrhoids | |  | |  | |  | |  | |  | |  | |  | |  | |  |
| vasodilators | | Peripheral artery disease | | titinnitus | | cognitive disorders | |  | |  | |  | |  | |  | |  | |  | |  |
| foot exam | | back pain | | knee pain | |  | |  | |  | |  | |  | |  | |  | |  | |  |
| Troponin testing | | Infarction diagnosis | |  | |  | |  | |  | |  | |  | |  | |  | |  | |  |
| biphsphonates | | all indications | | for more than 5 years | |  | |  | |  | |  | |  | |  | |  | |  | |  |
| transaminase testing | | as part of standard blood testing | | after a cholecystectomy | |  | |  | |  | |  | |  | |  | |  | |  | |  |
| Cardiology consultation | | As part of yearly checkup | | as part of a yearly checkup in patients older than 50 with low cardiovascular risk | |  | |  | |  | |  | |  | |  | |  | |  | |  |
| Otorhinolaryngology consultation | | mouth ulcer | | geographic tongue | |  | |  | |  | |  | |  | |  | |  | |  | |  |
| Synovial fluid analysis | | Hemarthrosis following trauma | | when the liquid is lemon yellow | |  | |  | |  | |  | |  | |  | |  | |  | |  |
| anatomic pathology analysis | | unsuspicious nevus | | Basal-cell carcinoma in patient older than 85 years | | skin tag | |  | |  | |  | |  | |  | |  | |  | |  |
| Gamma GT analysis | | in a liver check up | |  | |  | |  | |  | |  | |  | |  | |  | |  | |  |
| tramadol | | pain relief in the elderly | |  | |  | |  | |  | |  | |  | |  | |  | |  | |  |
| ultrasound imaging of the biliary tract | | dyspepsia | | right upper quadrant pain | | epigastric pain without improvement after using PPI (Proton pump inhibitors) | |  | |  | |  | |  | |  | |  | |  | |  |
| corticosteroid injections | | tendinitis | |  | |  | |  | |  | |  | |  | |  | |  | |  | |  |
| neuroleptic (or antipsychotic) | | sleep disorders | | mood disorders | |  | |  | |  | |  | |  | |  | |  | |  | |  |

**Appendix 3:** A sample summary


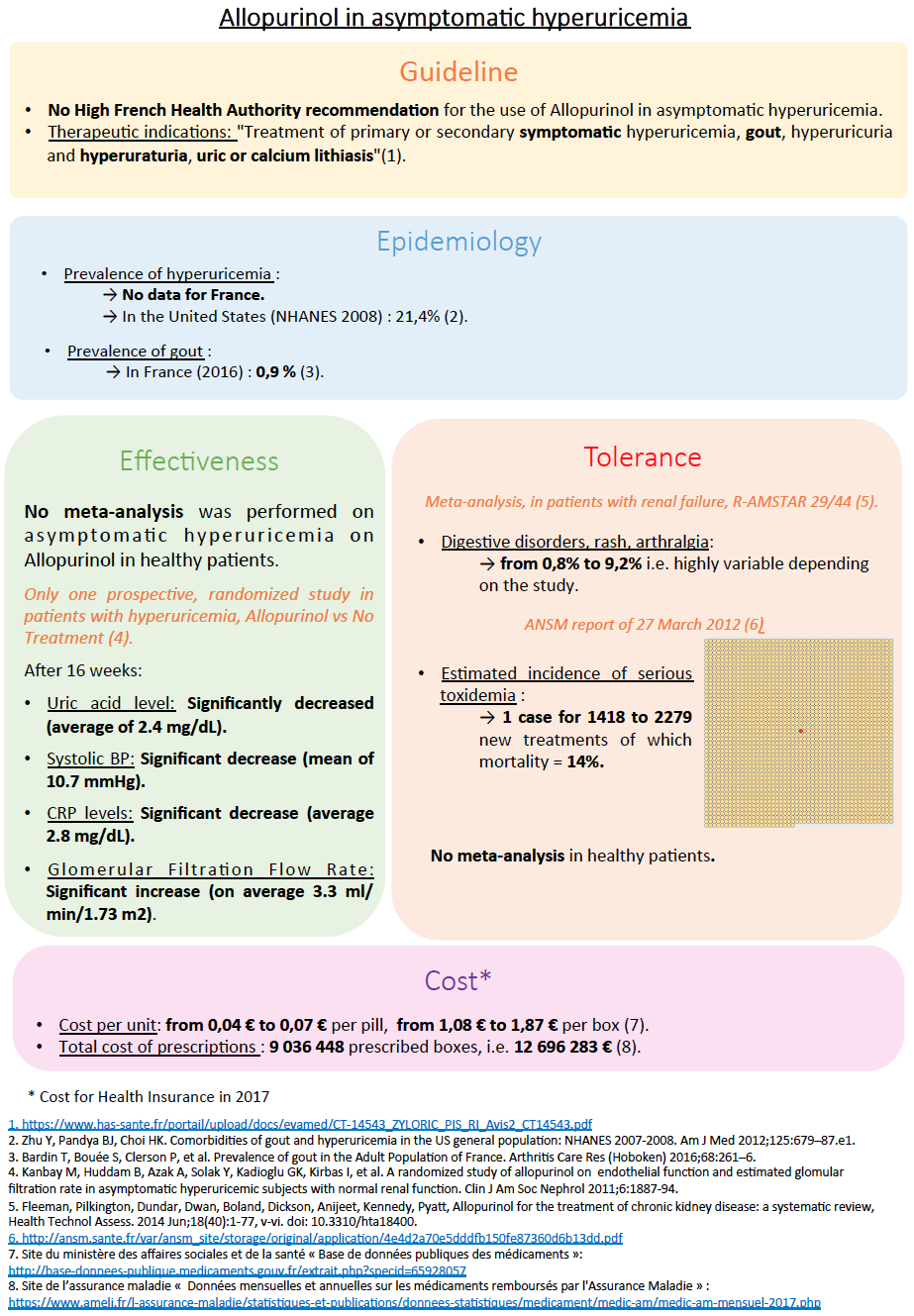


**Appendix 4:** Studies selected for summaries

| Item | Study selected | Type of score used | Score given |
| --- | --- | --- | --- |
| Systematic PSA testing in men older than 50 with no information given to the patient regarding the benefits and risks of the test | - Fenton JJ, Weyrich MS, Durbin S, Liu Y, Bang H, Melnikow J. Prostate-specific antigen–based screening for prostate cancer: evidence report and systematic review for the US Preventive Services Task Force. Jama. 2018 May 8;319(18):1914-31. | R-amstar | 38/44 |
| Antibiotics for acute bronchitis, nasopharyngitis, otitis media with effusion, or uncomplicated influenza | - Smith SM, Fahey T, Smucny J, Becker LA. Antibiotics for acute bronchitis. In: The Cochrane Collaboration, éditeur. Cochrane Database of Systematic Reviews. Chichester, UK: John Wiley & Sons, Ltd; 2017 | R-amstar | 40/44 |
|  | - Carrat F, Schwarzinger M, Housset B, Valleron A-J. Antibiotic treatment for influenza does not affect resolution of illness, secondary visits or lost workdays. Eur J Epidemiol. 2004;19(7):703?5. | Not applicable |  |
|  | - Venekamp RP, Burton MJ, van Dongen TM, van der Heijden GJ, van Zon A, Schilder AG. Antibiotics for otitis media with effusion in children. Cochrane ENT Group, éditeur. Cochrane Database Syst Rev | R-amstar | 39/44 |
|  | - Kenealy T, Arroll B. Antibiotics for the common cold and acute purulent rhinitis. In: The Cochrane Collaboration, éditeur. Cochrane Database of Systematic Reviews [Internet]. Chichester, UK: John Wiley & Sons, Ltd; 2013 | R-amstar | 40/44 |
| Prescription of statins in primary prevention of cardio-vascular risk in older patients | - Teng, M., Lin, L., Zhao, Y. J., Khoo, A. L., Davis, B. R., Yong, Q. W., ... & Lim, B. P. (2015). Statins for primary prevention of cardiovascular disease in elderly patients: systematic review and meta-analysis. Drugs & aging, 32(8), 649-661. | R-amstar | 41/44 |
| Prescription of antitussive or expectorants agents for acute cough or acute bronchitis care | - Smith SM, Schroeder K, Fahey T. Over-the-counter (OTC) medications for acute cough in children and adults in community settings. Cochrane Database of Systematic Reviews 2014, Issue 11. Art. No.: CD001831. DOI: 10.1002/14651858.CD001831.pub5. | R-amstar | 37/44 |
| Prescription of an homeopathic treatment (Influenzinum) for flu prevention | - Mathie RT, Lloyd SM, Legg LA, et al. Randomised placebo-controlled trials of individualised homeopathic treatment: systematic review and meta-analysis. Syst Rev 2014;3:142. | R-amstar | 34/44 |
|  | - Mathie RT, Ramparsad N, Legg LA, et al. Randomised, double-blind, placebo-controlled trials of non-individualised homeopathic treatment: systematic review and meta-analysis. Syst Rev 2017;6:63. | R-amstar | 36/44 |
|  | - Marinone C, Bastard M, Bonnet P-A, Gentile G, Casanova L. Efficacité d’un traitement préventif par Influenzinum en période hivernale contre la survenue d’un syndrome grippal. Thérapie 2017;72:465‐74. | Not applicable | Not applicable |
| Long term prescription of proton pump inhibitors without reviewing the indication | - Sigterman KE, van Pinxteren B, Bonis PA, et al. Short‐term treatmt with proton pump inhibitors, H2‐recept antagonists and prokinetics for gastro‐oesophageal reflux disease‐like symptoms and endoscop neg reflux disease. Cochrane DB of Systematic Reviews | R-amstar | 36/44 |
|  | - Khan M, Santana J, Donnellan C, Preston C, Moayyedi P. Medical treatments in the short term management of reflux oesophagitis. Cochrane Database Syst Rev. 2007 Apr 18;(2):CD003244. | R-amstar | 28/44 |
|  | - Rostom, A., Dube, C., Wells, G. A., Tugwell, P., Welch, V., Jolicoeur, E., ... & Lanas, A. (2002). Prevention of NSAID?induced gastroduodenal ulcers. Cochrane database of systematic reviews. | R-amstar | 39/44 |
|  | - Lambert AA, Lam JO, Paik JJ, Ugarte-Gil C, Drummond MB, Crowell TA. Risk of community acquired pneumonia with outpatient proton-pump inhibitor therapy: a systematic review and metaanalysis. PloS One. 2015;10(6):e0128004. | R-amstar | 38/44 |
|  | - Nochaiwong S, Ruengorn C, Awiphan R, et al. The association between proton pump inhibitor use and the risk of adverse kidney outcomes : a systematic review and meta-analysis. Nephrol Dial Transplant Off Publ Eur Dial Transpl Assoc - Eur Ren Assoc. 2018 Feb 23 | R-amstar | 39/44 |
|  | - Martin RM, Dunn NR, Freemantle S, Shakir S. The rates of common adverse events reported during treatment with proton pump inhibitors used in general practice in England: cohort studies. Br J Clin Pharmacol. 2000 Oct;50(4):366–72. | Not applicable |  |
| Prescription of a non-steroidal anti-inflammatory agent (NSAID) for the symptomatic treatment of acute sinusitis and pharyngitis | - Aspley, S., Shephard, A., Schachtel, E., Sanner, K., Savino, L., & Schachtel, B. (2016). Efficacy of flurbiprofen 8.75 mg lozenge in patients with a swollen and inflamed sore throat. Current medical research and opinion, 32(9), 1529-1538. | Not applicable |  |
|  | - Kim, S. Y., Chang, Y. J., Cho, H. M., Hwang, Y. W., & Moon, Y. S. (2013). Non?steroidal anti?inflammatory drugs for the common cold. Cochrane Database of Systematic Reviews, (6). | R-amstar | 40/44 |
| Prescription of DPP4 inhibitors in patients with type 2 diabetes | - Rehman MB, Tudrej BV, Soustre J, Buisson M, Archambault P, Pouchain D, et al. Efficacy and safety of DPP-4 inhibitors in patients with type 2 diabetes: Meta-analysis of placebo-controlled randomized clinical trials. Diabetes Metab. 2017 Feb;43(1):48–58. | R-amstar | 30/44 |
|  | - Scirica BM, Bhatt DL, Braunwald E, Steg PG, Davidson J, Hirshberg B, et al. Saxagliptin and Cardiovascular Outcomes in Patients with Type 2 Diabetes Mellitus. N Engl J Med. 3 oct2013;369(14):1317?26. | Not applicable |  |
|  | - White WB, Cannon CP, Heller SR, Nissen SE, Bergenstal RM, Bakris GL, et al. Alogliptin after acute coronary syndrome in patients with type 2 diabetes. N Engl J Med. 3 oct 2013;369(14):1327?35 | Not applicable |  |
|  | - Green JB, Bethel MA, Armstrong PW, Buse JB, Engel SS, Garg J, et al. Effect of Sitagliptin on Cardiovascular Outcomes in Type 2 Diabetes. N Engl J Med. 16 juill 2015;373(3):232?42. | Not applicable |  |
| Prescription of allopurinol for asymptomatic hyperuricemia in prevention of gout attack, renal hypertension, cardio-vascular disease | - Kanbay M, Huddam B, Azak A, et al. A randomized study of allopurinol on endothelial function and estimated glomular filtration rate in asymptomatic hyperuricemic subjects with normal renal function. Clin J Am Soc Nephrol 2011;6:1887-94 | Not applicable |  |
|  | - Fleeman, Pilkington, Dundar, Dwan, Boland, Dickson, Anijeet, Kennedy, Pyatt, Allopurinol for the treatment of chronic kidney disease: a systematic review, Health Technol Assess. 2014 Jun;18(40):1-77, v-vi. doi: 10.3310/hta18400. | R-amstar | 29/44 |
|  | - AFSSAPS. Commission nationale de pharmacivigilance - Compte rendu de la réunion du 27 mars 2012 | Not applicable |  |
| Systematic screening using mammography for breast cancer in women with no information given to the patient regarding the benefits and risks | - Gøtzsche PC, Jørgensen KJ. Screening for breast cancer with mammography. In: Cochrane Database of Systematic Reviews. John Wiley & Sons, Ltd; 2013. | R-amstar | 39/44 |
|  | - Nelson HD, Cantor A, Humphrey L, Fu R, Pappas M, Daeges M, et al. Screening for Breast Cancer: A Systematic Review to Update the 2009 U.S. Preventive Services Task Force Recommendation. Rockville (MD): Agency for Healthcare Research and Quality (US); 2016 | R-amstar | 39/44 |
| Prescription of cholinesterase inhibitors and memantin for mild cognitive impairment and Alzheimer’s disease | - T. Russ, The Cochrane Collaboration, 2012 « Cholinesterase inhibitors for mild cognitive impairment » | R-amstar | 41/44 |
|  | - H. Kobayashi, International Journal of Psychiatry, 2015 « The comparative efficacy and safety of cholinesterase inhibitors in patients with mild-to-moderate Alzheimer’s disease: a Bayesian network meta-analysis » | R-amstar | 32/44 |
|  | - S. Matsunaga, PLoS ONE, 2015 « Memantine monotherapy for Alzheimer’s disease: a systematic review and meta-analysis » | R-amstar | 40/44 |
| Benzodiazepine prescription for generalised anxiety, benzodiazepine prescription for all indication in older patients, prescription of hypnotics for insomnia | - Bandelow, B., Reitt, M., Röver, C., Michaelis, S., Görlich, Y., & Wedekind, D. (2015). Efficacy of treatments for anxiety disorders: a meta-analysis. International Clinical Psychopharmacology, 30(4), 183-192 | R-amstar | 33/44 |
|  | - Riemann D, Voderholzer U, Cohrs S, Rodenbeck A, Hajak G, Rüther E, et al. Trimipramine in primary insomnia: results of a polysomnographic double-blind controlled study. Pharmacopsychiatry. sept 002;35(5):165‑74. | Not applicable |  |
|  | - Buscemi, N., Vandermeer, B., Friesen, C., Bialy, L., Tubman, M., Ospina, M., ... & Witmans, M. (2007). The efficacy and safety of drug treatments for chronic insomnia in adults: a meta-analysis of RCTs. Journal of general internal medicine, 22(9), 1335. | R-amstar | 33/44 |
|  | - Brasure M, MacDonald R, Fuchs E, Olson CM, Carlyle M, Diem S, et al. Management of Insomnia Disorder [Internet]. Rockville (MD): Agency for Healthcare Research and Quality (US); 2015. (AHRQ Comparative Effectiveness Reviews). | R-amstar | 29/44 |
|  | - Wilt TJ, MacDonald R, Brasure M, Olson CM, Carlyle M, Fuchs E, et al. Pharmacologic Treatment of Insomnia Disorder: An Evidence Report for a Clinical Practice Guideline by the American College of Physicians. Ann Intern Med. 19 juill 2016;165(2):103‑12. | R-amstar | 34/44 |
|  | - Parsaik, A. K., Mascarenhas, S. S., Khosh-Chashm, D., Hashmi, A., John, V., Okusaga, O., & Singh, B. (2016). Mortality associated with anxiolytic and hypnotic drugs—A systematic review and meta-analysis. Australian & New Zealand Journal of Psychiatry, 50(6):520-33 | R-amstar | 34/44 |
|  | - Xing D, Ma XL, Ma JX, Wang J, Yang Y, Chen Y. Association between use of benzodiazepines and risk of fractures: a meta-analysis. Osteoporosis International. 2014 Jan 1;25(1):105-20. | R-amstar | 33/44 |
|  | - Elvik, R. (2013). Risk of road accident associated with the use of drugs: a systematic review and meta-analysis of evidence from epidemiological studies. Accident Analysis & Prevention, 60, 254-267. | R-amstar | 33/44 |
|  | - Lader, M. (2011). Benzodiazepines revisited—will we ever learn?. Addiction, 106(12), 2086-2109. | Not applicable |  |
| Imaging for low back pain evolving for less than 6 weeks | - Chou, R., Fu, R., Carrino, J. A., & Deyo, R. A. (2009). Imaging strategies for low-back pain: systematic review and meta-analysis. The Lancet, 373(9662), 463-472. | R-amstar | 35/44 |
| Prescription of vasodilator agents in the treatment of peripheral Arterial Disease | - De Backer TLM, Vander Stichele R, Lehert P, Van Bortel L. Naftidrofuryl for intermittent claudication. Cochrane Database Syst Rev. 16 avr 2008;(2):CD001368. | R-amstar | 39/44 |
|  | - Nicolaï SPA, Kruidenier LM, Bendermacher BLW, Prins MH, Stokmans RA, Broos PPHL, et al. Ginkgo biloba for intermittent claudication. Cochrane Database Syst Rev. 6 juin 2013;(6):CD006888. | R-amstar | 36/44 |
| Prescription of tramadol or tramadol with paracetamol in the elderly | - Vorsanger G, Xiang J, Jordan D, Farrell J. Post Hoc Analyss of Randomzd, Double-Blind, Placebo-Controlled Efficacy and Tolerability Study of Tramadol Extended Release for the Treatmt of Osteoarthritis Pain in Geriatric Patients. Clin Ther. 2007:29(11) | Not applicable |  |
